# Acyl-coenzyme A: cholesterol acyltransferase inhibitor avasimibe suppresses tumorigenesis and induces G1-phase cell-cycle arrest by activating PPARγ signaling pathway in bladder cancer

**DOI:** 10.7150/jca.83856

**Published:** 2024-01-01

**Authors:** Tianchen Peng, Kangping Xiong, Zhiwen He, Songtao Cheng, Siming Chen, Song Chen, Zhonghua Yang, Wan Xiang, Lingao Ju, Yi Zhang, Kaiyu Qian, Yu Xiao, Gang Wang

**Affiliations:** 1 Department of Urology, Hubei Key Laboratory of Urological Diseases, Zhongnan Hospital of Wuhan University, Wuhan, China.; 2 Department of Biological Repositories, Zhongnan Hospital of Wuhan University, Wuhan, China.; 3 Human Genetic Resource Preservation Center of Hubei Province, Wuhan, China.; 4 Laboratory of Precision Medicine, Zhongnan Hospital of Wuhan University, Wuhan, China.; 5 Euler Technology, ZGC Life Sciences Park, Beijing, China.; 6 Center for Quantitative Biology, School of Life Sciences, Peking University, Beijing, China.

**Keywords:** Bladder cancer (BLCA), acyl-coenzyme A: cholesterol acyltransferase-1 (ACAT1), avasimibe, PPARγ, cell cycle.

## Abstract

Reprogramming of energy metabolism is one of the most important characteristics of tumors. Bladder cancer (BLCA) cells contain higher levels of cholesterol content compared to normal cells, and acyl-coenzyme A (CoA): cholesterol acyltransferase-1 (ACAT1) plays a crucial role in the esterification of cholesterol. Avasimibe is a drug that has been used in the treatment of atherosclerosis, and it can effectively inhibit ACAT1. We observed that ACAT1 was significantly up-regulated in BLCA and positively correlated with tumor grade. By avasimibe administration, the proliferation and migration ability of BLCA cells were reduced, while the production of ROS was strongly increased, accompanied by the up-regulated expression of ROS metabolism-related proteins SOD2 and catalase. Furthermore, BLCA cell cycle was arrested at the G1 phase, accompanied by the downregulation of cell cycle-related proteins (CCNA1/2, CCND1, CDK2 and CDK4), while the PPARγ was found to be up-regulated at both transcriptional and protein levels after avasimibe treatment. Then we found that the PPARγ antagonist GW9662 could reverse the effect of avasimibe on the cell cycle. Moreover, xenograft and pulmonary metastasis models further demonstrated that avasimibe could inhibit tumor cell growth and metastasis *in vivo*. Taken together, our results for the first time revealed that avasimibe can inhibit BLCA progression and metastasis, and PPARγ signaling pathway may play a key role in regulation of cell cycle distribution induced by avasimibe.

## Introduction

Reprogramming of energy metabolism is one of the most important characteristics of tumors 1, and changes in lipid metabolism (including cholesterol metabolism) are also considered to be a new characteristic for malignant cancer 2. Because of the remarkable distinction compared to the normal cells, tumor metabolic pathways are considered potential new targets for selective chemotherapy 3. Cancer cells maintain high intracellular cholesterol levels by taking up low-density lipoprotein (LDL) and *de novo* synthesis of large amounts of cholesterol 4. However, the cholesterol esterification process mediated by acyl-coenzyme A: cholesterol acyltransferase-1 (ACAT1) was able to avoid cytotoxicity caused by high intracellular levels of cholesterol 5.

ACAT1 is a membrane-bound enzyme that regulates cholesterol metabolism 6, converting free cholesterol to cholesteryl esters and attenuates lipid raft formation 7. Inhibition of ACAT1 has been documented to suppresses pancreatic cancer cell growth, mainly due to increasing intracellular free cholesterol levels, which can increase endoplasmic reticulum (ER) stress and ultimately lead to apoptosis 8. In addition, ACAT1 inhibition has been shown to be effective in suppressing the proliferation and migration of hepatocellular carcinoma cells 9. Thus, ACAT1 has the potential to be a therapeutic target in anti-tumor therapy. Avasimibe is a small molecule drug that can effectively inhibit ACAT1. Not only has it been used clinically to treat atherosclerosis 10, but also has been reported to play an essential role in cancer immunotherapy by elevating TCR signaling levels in CD8^+^ T cells 11. However, the expression of ACAT1 in BLCA as well as whether its inhibitor (avasimibe) can suppress the growth of BLCA has not been reported yet.

One of the high-risk factors of BLCA is dietary lipid (including cholesterol) intake 12. Recently, our group has conducted several studies on lipid metabolism of BLCA 13, 14. Simvastatin, a widely used statin, can activate PPARγ-mediated signaling, inducing cell viability inhibition and cell cycle arrest 14. It comes to be a conclusion that PPARγ can modulate important regulatory pathways related to lipid metabolism in BLCA 15. Clinically, BLCA is usually divided into two major subtypes: muscle-invasive bladder cancer (MIBC) and non-muscle-invasive bladder cancer (NMIBC), which have very different clinical manifestations and tumor biological behaviors 16, 17. However, studies reported that compared to NMIBC cells RT4, the cholesterol levels were higher in MIBC T24 cells 18. Therefore, we chose MIBC cells 5637 and T24 for our experiments. In this study, we verified that ACAT1 was significantly up-regulated in BLCA and positively correlated with tumor grade, and the inhibitory effect of avasimibe on BLCA may be related to the abnormal activation of PPARγ signaling pathway, revealing that the inhibition of ACAT1 by avasimibe may provide a new idea to study the potential treatments or adjuvant therapy for BLCA.

## Materials and methods

### Human BLCA tissue samples

The BLCA (stage II) tissue samples (n = 10) and matched paracancerous tissue samples (n = 10) were obtained from male patients (ages 65.60 ± 6.99) with BLCA after radical cystectomy at Zhongnan Hospital of Wuhan University. Inclusion criteria were BLCA patients requiring radical cystectomy, without metabolism-related diseases, and not taking lipid-lowering drugs. Exclusion criteria included patients with BLCA in combination with other tumors, with metabolic diseases such as hyperlipidemia and diabetes mellitus, taking lipid-lowering drugs, and patients with secondary bladder tumors. Informed consent was obtained from all subjects to collect the samples, and it was conducted in accordance with the Declaration of Helsinki. This study was approved by the Ethics Committee of Zhongnan Hospital of Wuhan University (approval number: 2017050). The sample collection and treatment procedures were conducted in accordance with the approved guidelines.

### Bioinformatics analysis

ACAT1 mRNA expression data in 411 BLCA tissues and 19 normal bladder tissues were obtained from the TCGA database, along with the corresponding clinical data of the patients (Supplementary Table S1). Patients were classified as high ACAT1 expression and low ACAT1 expression based on the average expression of all BLCA patients. Patients' overall survival curves and disease-free survival curves were calculated using the Kaplan-Meier method and clinical correlation analysis was performed by corresponding clinical data. To explore the function of ACAT1 in BLCA progression, we performed gene set enrichment analysis (GSEA, *http://software.broadinstitute.org/gsea/index.jsp*) 19.

### BLCA cells culture

The human MIBC cell lines, 5637 (Cat. #TCHu1) and T24 (Cat. #SCSP-536), human uroepithelial cells SV-HUC-1 (Cat. #TCHu169), and human renal tubular epithelial cells HK2 (Cat. #GNHu47) were both obtained from the Chinese Academy of Sciences (Shanghai, China) and have been identified by the China Centre for Type Culture Collection (Wuhan, China). 5637, T24 and SV-HUC-1 cells were cultured with RPMI-1640 medium (containing 10% fetal bovine serum) in a 37 ºC incubator with 5% CO_2_. HK2 cells were cultured with MEM medium (containing 10% fetal bovine serum) in a 37 ºC incubator with 5% CO_2_.

### Avasimibe treatment for BLCA cells

Cells were seeded into 6-well plates and cultured for 24 hrs, then treated with avasimibe (MCE, USA) at three concentrations: 0, 10 and 20 μM. After 48 hrs of drug treatment, cells were collected for cell phenotype experiments or detected with flow cytometric analysis or Western blots analysis.

### MTT assay

200 μL medium suspended with 3,000 cells was transferred into each well of 96-well plate and treated with avasimibe after 24 hrs. After 48 hrs of drug treatment, 20 μL 5 mg/mL MTT was added. Medium was removed from the 96-well plate after 4 hrs incubation, then 150 μL DMSO was added to each well. Shake gently on a shaker for 10 min until the formazan precipitate is fully dissolved. Finally, the absorbance of each well at 570 nm was tested using a microplate reader (Molecular Devices, USA) to assess cell viability.

### Clonogenic survival assay

2 μL medium suspended with 1,000 cells were seeded in each well of 6-well plates and cultured until the colonies emerged and grew to the appropriate size. After removing the medium, the cells were fixed and stained with 4% paraformaldehyde and 0.1% crystal violet solution sequentially. Cell viability was judged according to the number of clones in each well.

### Wound healing assay

Traces in the six-well plate were scratched with a 200 μL tip, when the cells grew to a very high density. Then cells were washed twice with PBS, followed by the addition of medium with different concentrations of avasimibe. At 0 hr and 24 hr after drug treatment, the results were photographed at several pre-labeled points with a phase contrast microscope, respectively. Finally, the gap closure was statistically analyzed.

### Transwell chamber migration assay

After drug treatment for 48 hrs, a total of 1.2 × 10^5^ 5637 cells, 5 × 10^4^ T24 cells, 1× 10^6^ SV-HUC-1 cells or 4 × 10^4^ HK2 cells are suspended in 200 μL medium without serum. The suspension was seeded in the upper transwell chamber, and 600 μL medium containing 10% fetal bovine serum was seeded into the lower chamber. Differences in nutrients between the upper and lower medium could induce cell migration. After incubation at 37°C for 24 hrs, cells were fixed and stained with 4% paraformaldehyde and 0.1% crystal violet solution sequentially. Then a phase contrast microscope was used to photograph and count cells that has migrated.

### Flow cytometry analysis for reactive oxygen species (ROS) and cell cycle

For testing ROS production, fluorescent probe 2',7'-Dichlorofluorescin diacetate (DCFH-DA) was used as a dye for ROS production. After treated with avasimibe for 48 hrs, cells were collected and washed with serum-free medium, then resuspended in serum-free medium containing 10 mM DCFH-DA (Sigma, USA). After 30 min incubation in 37°C, cells were washed again for three times using medium without serum. Finally, the levels of intracellular ROS production were measured by flow cytometry (Beckman, USA).

For cell cycle analysis, the collected cells were washed twice with cold PBS. After recentrifugation, the supernatant was removed and the cells were resuspended with 1X DNA staining solution and permeabilization solution (Multi sciences, China). After 30 min incubation in the dark, the cell cycle distribution of each group was analyzed by flow cytometry.

### Western blot analysis

BLCA cells were collected and lysed in RIPA buffer (containing protease inhibitor and phosphatase inhibitor) on ice for 30 min. The cell lysates were centrifuged at 12,000 g for 15 min and the supernatant was collected. For Western blot, total protein was separated using 7.5-12.5% SDS-PAGE gels, then transferred to PVDF membrane (Millipore, USA). Membranes were sealed in 5 % TBST fat-free milk for 2 hrs at room temperature. The membranes were cut horizontally according to the protein marker (Shanghai Epizyme, China) on the membrane and incubated separately with the appropriate primary antibody (Supplementary Table S2) for overnight at 4°C. After washing three times with TBST, membranes were incubated with secondary antibody (Supplementary Table S2) for 2 hrs at room temperature. Bands were detected using an enhanced chemiluminescence kit (Bio-Rad, USA) and blots were exposured to the BioSpectrum Gel Doc-IT2 315 167 Imaging System (UVP, USA).

### Quantitative reverse transcription PCR (qRT-PCR)

HiPure Total RNA Mini Kit (Magen, China) was utilized to extract total RNA from bladder samples and cell lines. The ReverTra Ace qPCR RT Kit (Toyobo, Japan) was used for the reverse transcription. 500 ng cDNA templates were added to a PCR system with a final volume of 20 μL. The primer sequences are listed in Supplementary Table S3.

### Luciferase reporter assay

PPARγ-Luc Reporter Plasmid was purchased from Genomeditech Co. Ltd (Shanghai, China). The luciferase reporter assay was performed with Luc-Pair™ Duo-Luciferase Assay Kit 2.0 (GeneCopoeial Inc, USA) according to its protocol.

### Immunofluorescence staining

Avasimibe treated BLCA cells were plated on 12-mm coverslips and incubated for 12 hrs. After the cells adhered, the coverslips were washed twice with PBS, then fixed with 4% paraformaldehyde for 30 min, washed with PBS again and then soaked with PBS. The rest of the processes were performed by Biofavor Biotech Co. Ltd (Wuhan, China).

### Hematoxylin and Eosin (H&E) Staining

Samples were processed with xylene, graded alcohol (100%, 96%, 80%, 70% ethanol) and H_2_O in turn. Then 10% hematoxylin (Sigma-Aldrich, USA) was used for variegation for 7 min and washed with water. 1% eosin (Sigma‐Aldrich, USA) and 0.2% glacial acetic acid was applied to highlight the cytoplasm for only seconds and washed with water again. Then samples were dehydrated in graded alcohol (70%, 80%, 96%, 100% ethanol) and xylene in turn. Finally, an inverted phase contrast microscope (Leica, Germany) was used for taking photos.

### Xenograft model and pulmonary metastasis model

3-week-old male BALB/c-nude mice were purchased from Beijing Vital River Laboratory Animal Technology Co. Ltd (Beijing, China). Before the experiment was performed, mice were maintained for one week for adaptation in an SPF-grade environment in the animal experiment center of Zhongnan Hospital of Wuhan University and approved by the institutional Experimental Animal Welfare Ethics Committee (approval No. ZN2021254). The xenograft model was established by subcutaneous injection of 150 μL PBS solution, containing 2 × 10^7^ of T24 cells, in the dorsal region near the forelimb of mice. Twelve days later, mice were randomly divided into two groups (n = 6). The experimental group was treated with avasimibe, which was dissolved in PBS containing 1% Tween 80, intraperitoneally at a dose of 30 mg/kg every other day, while the control group was similarly intraperitoneally injected with 1% Tween80 in PBS. The tumor size was measured with a vernier caliper and calculated according to the formula (tumor size = length × width^2^ × 0.5 mm^3^) at regular intervals. After 35 consecutive doses, the mice were sacrificed by cervical dislocation, and the tumors were collected. After the weight was measured, tumor tissues were fixed in 4% paraformaldehyde in preparation for subsequent staining.

For the pulmonary metastasis model, 100 μL of PBS solution containing 1 × 10^6^ T24 cells was injected into tail vein of mice. After 5-week-drug-treatment as in the xenograft model, the fluorescence of BLCA cells was observed using a Fusion FX7 Spectra Imaging system (Vilber, France), and the mice were subsequently sacrificed by cervical dislocation, and the lung tissues were removed and fixed with 4% paraformaldehyde for subsequent staining.

### Statistical analyses

All experiments were stable with at least three replicates. GraphPad Prism Version 8.0 was used for statistical analysis. To assess statistical differences of experimental results, two-tailed Student's T-test were used. *p* < 0.05 indicates significant difference in data. Levels of statistical significance are indicated as follows: *: *p* < 0.05; **: *p* < 0.01; ***: *p* < 0.001; ns: not significant (*p* > 0.05).

## Results

### ACAT1 was highly expressed in BLCA tissues and was associated with high pathological grade and poor clinical prognosis

**Figure 1:**
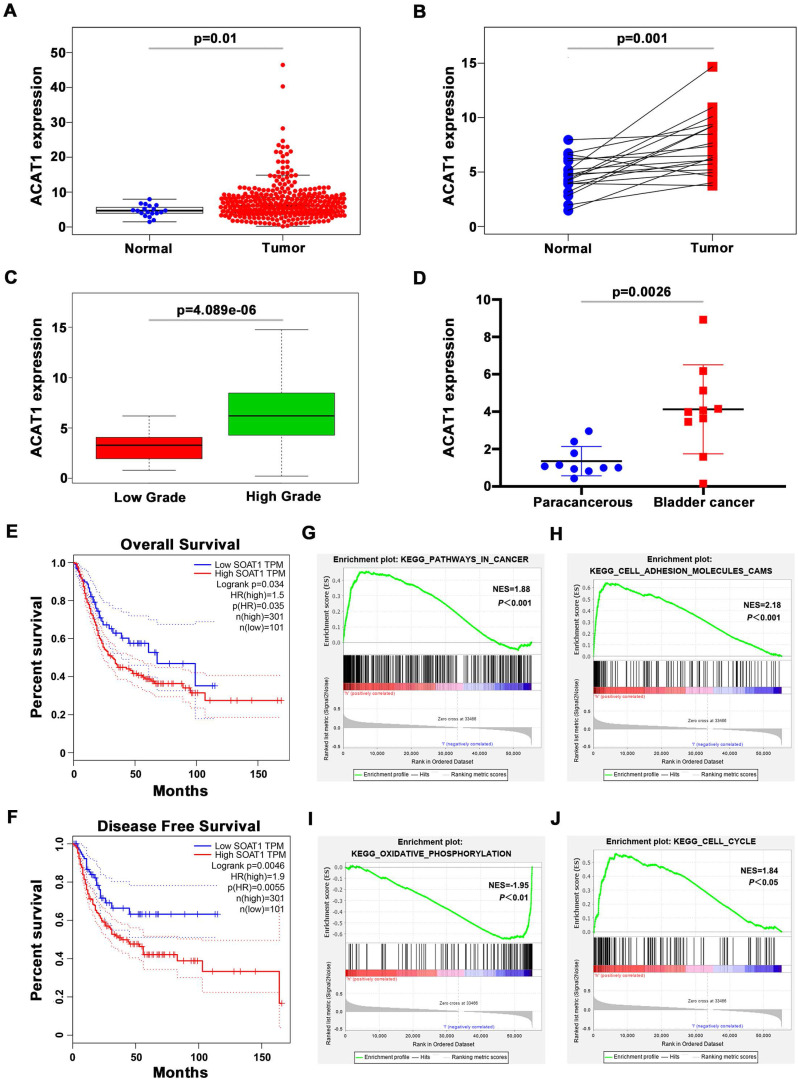
** ACAT1 was highly expressed in BLCA patients and was associated with high pathological grade and poor clinical prognosis. (A-B)** Analysis of ATAC1 mRNA expression in BLCA unpaired (A) / paired (B) samples based on TCGA database.** (C)** Correlation between ATAC1 expression levels and BLCA pathological grade. **(D)** qRT-PCR analysis of the expression of PPARγ mRNA in BLCA (n = 10) and matched paracancerous tissues (n = 10). **(E-F)** Kaplan-Meier survival analysis of BLCA patients based on TCGA database, overall survival (E) and disease-free survival (F). **(G-J)** GSEA emphasized the correlation between increased ACAT1 expression and pathways in cancer (G), cell adhesion molecules cams (H), oxidative phosphorylation (I), and cell cycle (J).

We first evaluated the TCGA database and performed corresponding bioinformatics analysis on its 411 BLCA samples and 19 normal bladder samples. The clinicopathological features of the patients in TCGA database were shown in Supplementary Table S1. In unpaired samples, we found that ACAT1 expression in BLCA specimens was significantly higher than normal specimens (Figure 1A). The same conclusion was validated in the paired BLCA and paraneoplastic tissues (Figure 1B). And the expression of ACAT1 had a significant positive correlation with the pathological grade of BLCA (Figure 1C). We further extracted total RNA from 10 pairs of BLCA tissues and matched paracancerous tissues, and the qRT-PCR showed that mRNA expression of ACAT1 was significantly up-regulated in BLCA (Figure 1D). In addition, Kaplan-Meier survival analyses showed that patients with high ACAT1 expression were more pessimistic than those with low expression in both overall survival (Figure 1E) and disease-free survival (Figure 1F). GSEA revealed that ACAT1 was functionally enriched in pathways in cancer (Figure 1G), cell adhesion molecules cams (Figure 1H), oxidative phosphorylation (Figure 1I), and cell cycle (Figure 1J).

### Avasimibe inhibited BLCA cells viability and proliferation

**Figure 2:**
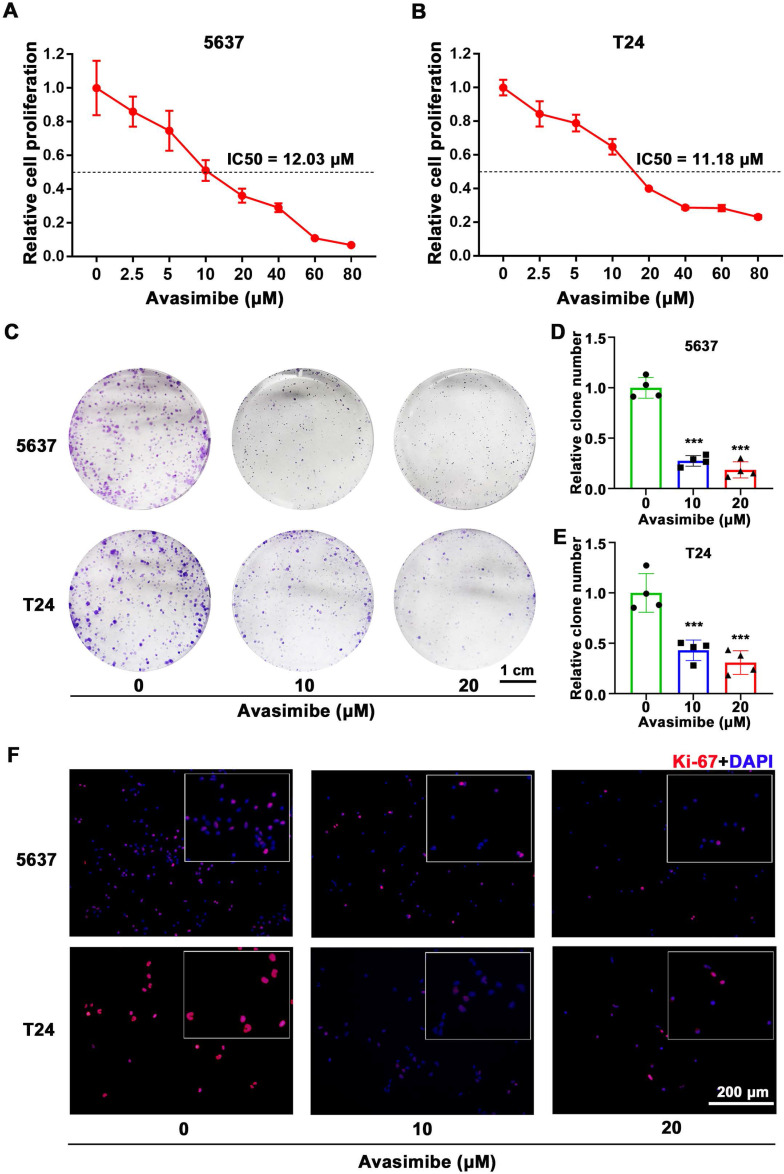
** Avasimibe inhibited BLCA cells viability and proliferation. (A-B)** MTT assay was used to test the viability of 5637 (A) and T24 (B) cells treated with avasimibe. **(C)** Influence of avasimibe on cell survival was detected by clonogenic survival assay. **(D-E)** The statistical diagram of clonogenic formation assay, ***: *p* < 0.001.** (F)** Immunofluorescence staining of Ki-67 (Red) in BLCA cells with/without avasimibe treatment. Nuclei were stained by DAPI (blue). The scale bar is 200 μm.

To study the possible inhibitory effect of avasimibe on BLCA cells, 5637 and T24 cells were treated with a gradient concentration of avasimibe. After 48 hrs of drug treatment, the cell proliferation ability was determined by MTT assay. As the result showed, the proliferation ability of 5637 cells (IC50 is 12.03 μM, Figure 2A) and T24 cells (IC50 is 11.18 μM, Figure 2B) were both inhibited by avasimibe in a dose-dependent manner. In addition, compared with the human uroepithelial cells SV-HUC-1 (IC50 is 47.78 μM, Supplementary Figure S1A) and human renal tubular epithelial cells HK2 (IC50 is 57.54 μM, Supplementary Figure S1B), BLCA cells were more sensitive to avasimibe. Subsequent clonogenic formation assay also determined the inhibitory effect of avasimibe on viability of BLCA cells (Figure 2C-E), whereas the inhibitory effect was not significant in SV-HUC-1 and HK2 cells (Supplementary Figure S1C-D). Furthermore, the Ki-67 staining of BLCA cells treated with avasimibe was decreased obviously (Figure 2F), indicating that the tumorigenesis ability of the cells was inhibited by avasimibe.

### Avasimibe suppressed BLCA cells migration

**Figure 3:**
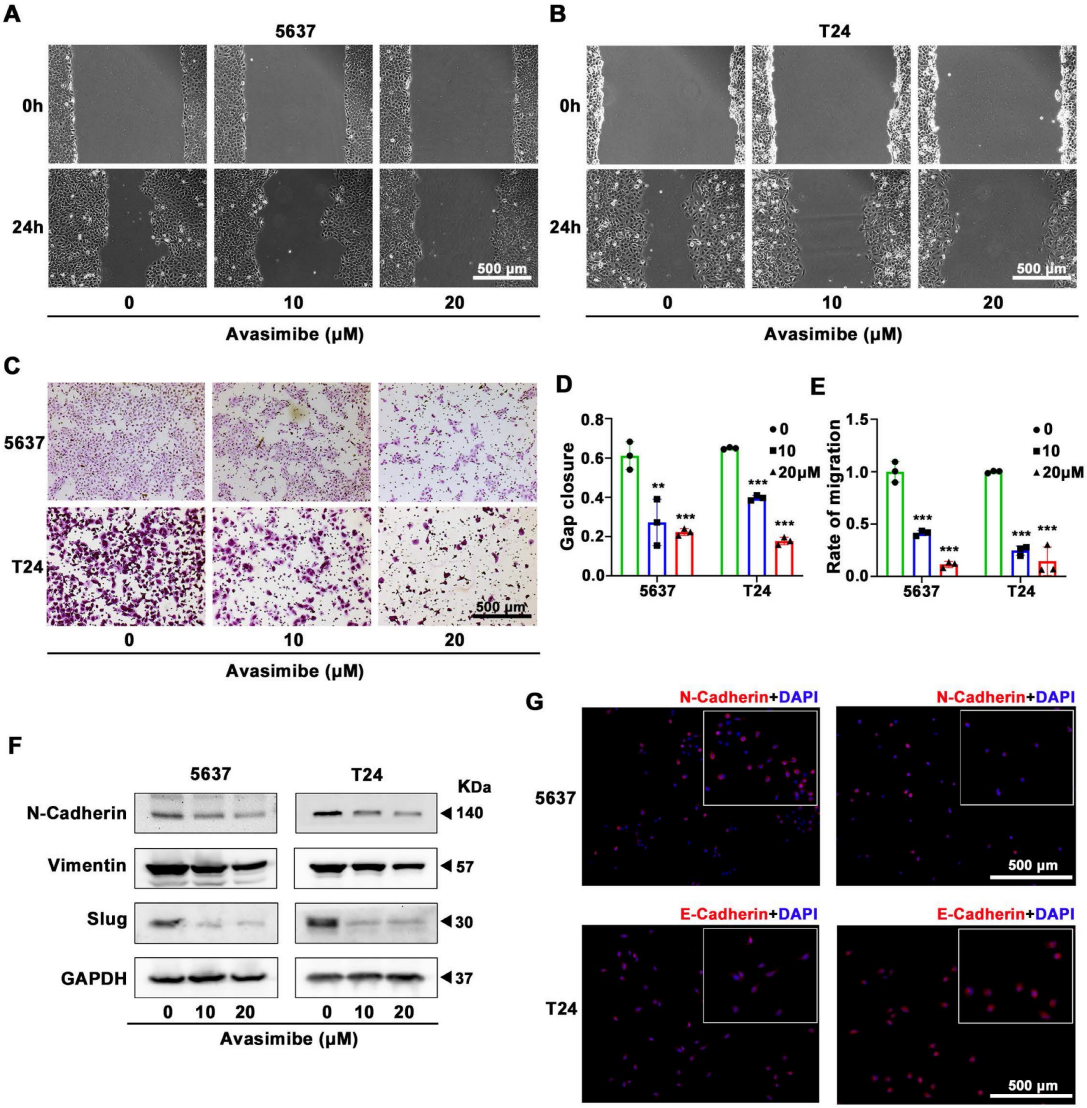
** Avasimibe suppressed BLCA cells migration. (A-B)** Wound healing assay was used to test the migration of 5637 (A) and T24 (B) after avasimibe treatment. **(C)** The influence of avasimibe on cell migration was detected by transwell chamber migration assay. **(D)** Statistical diagram of wound healing assay, **: *p* < 0.01; ***: *p* < 0.001. **(E)** The statistical diagram of transwell chamber migration assay. **(F)** Western blot analysis was used to evaluate the content of proteins associated with EMT: N-cadherion, Vimention and Slug. **(G)** Immunofluorescence staining of N-cadherion (Red) in 5637 cells and E-cadherion (Red) in T24 cells. Nuclei were stained by DAPI (blue). The scale bar is 500 μm.

To study the antimetastatic ability of avasimibe, wound healing migration assay was conducted. Both 5637 (Figure 3A) and T24 (Figure 3B) cells showed reduced migration capacity after treated with avasimibe, and the inhibitory effect rose with the increase of drug concentration (Figure 3D). To eliminate the interference caused by cell proliferation, we further performed the transwell migration assay to detect the effect of avasimibe on the migration ability of BLCA cells. Results showed that the migration ability of avasimibe-treated BLCA cells were inhibited (Figure 3C and 3E). In contrast, the migration ability of SV-HUC-1 and HK2 cells was not significantly inhibited after avasimibe treatment (Supplementary Figure S2A-F). Epithelial-Mesenchymal Transition (EMT) is closely related to tumor metastasis. The Western blot results showed that the expression levels of EMT-related proteins (N-cadherin, Vimentin and Slug) were significantly decreased (Figure 3F). Subsequent immunofluorescence-based analysis confirmed above result, showing that the N-cadherin-derived fluorescence intensity was significantly reduced in avasimibe treated 5637 cells, while the E-cadherin-derived fluorescence intensity was remarkably increased in avasimibe treated T24 cells (Figure 3G).

### Avasimibe increased ROS stress and induced cell cycle arrest

**Figure 4:**
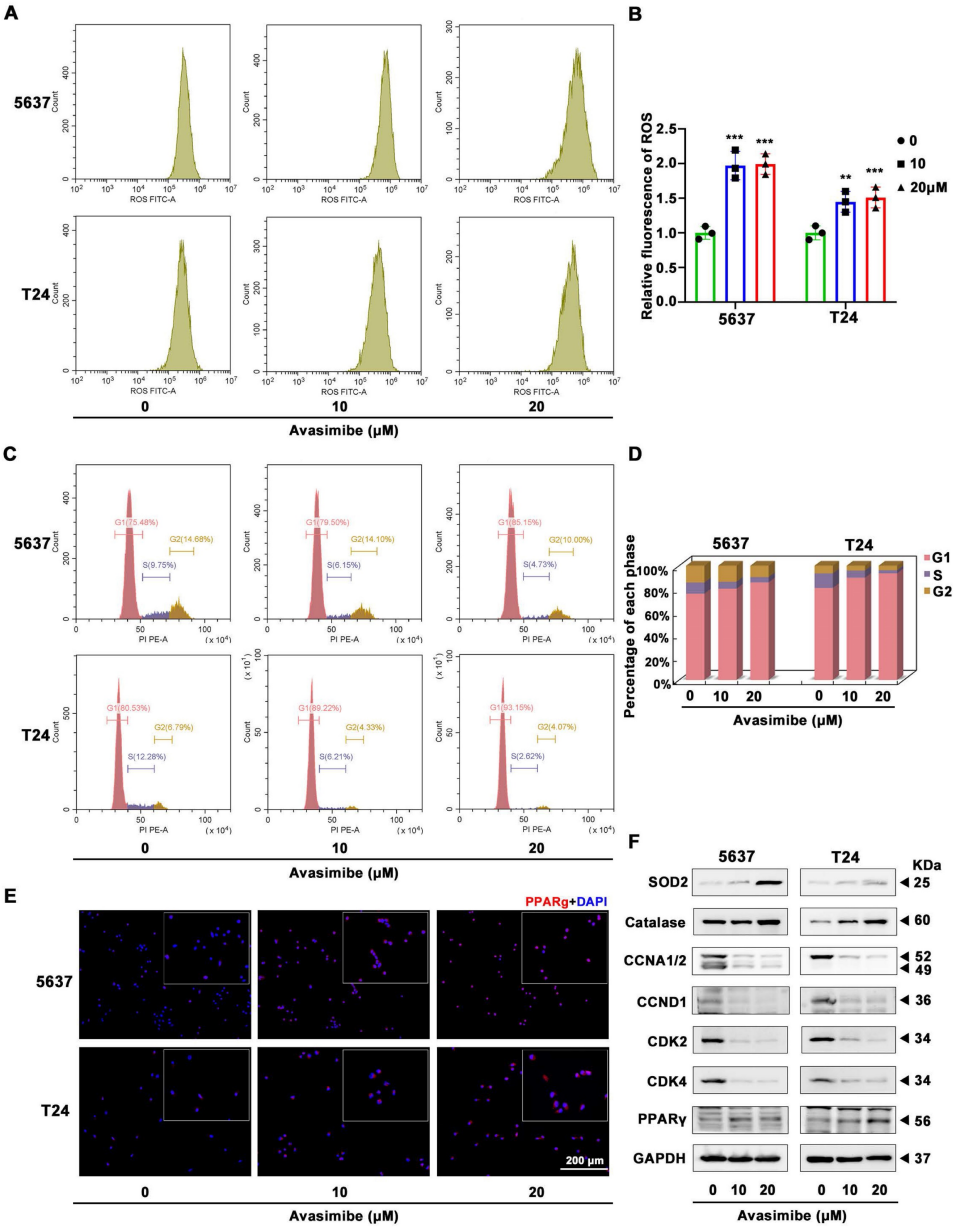
** Avasimibe increased ROS stress and induced cell cycle arrest accompanied by activated PPARγ signaling pathway. (A-B)** The production of ROS was detected by flow cytometry analysis and the statistical diagram, **: *p* < 0.01; ***: *p* < 0.001. **(C-D)** The cell cycle distribution of BLCA cells after avasimibe treatment was analyzed by flow cytometry. **(E)** Immunofluorescence staining of PPARγ (Red) in BLCA cells with/without avasimibe treatment. Nuclei were stained by DAPI (blue). The scale bar is 200 μm. **(F)** Western blot analysis was used to evaluate the content level of proteins associated with ROS, cell cycle and PPARγ.

Flow cytometry analysis was used to assess ROS stress level and showed a significant increase after avasimibe administration (Figure 4A-B). Meanwhile, we applied flow cytometry to detect the effect of avasimibe on cell cycle distribution. The result showed that after avasimibe treatment, the cell cycle was obviously arrested at the G1 phase (Figure 4C-D). In contrast, in SV-HUC-1 and HK2 cells, avasimibe did not cause significant changes in ROS stress levels (Supplementary Figure S3A-C). Moreover, HK2 cells arrested at the G1 phase only when avasimibe was administered at a concentration of 40 μM (Supplementary Figure S3D-F). The expression levels of proteins involved in ROS stress (catalase and SOD2) and cell cycle regulation (CCNA1/2, CCND1, CDK2 and CDK4) were analyzed by Western blot. The results exhibited an upregulation of catalase and SOD2, and a downregulation of CCNA1/2, CCND1, CDK2 and CDK4 in the BLCA cells after avasimibe treatment (Figure 4F). In addition, it was found that the PPARγ protein level was significantly increased after avasimibe treatment (Figure 4F), which was also confirmed by immunofluorescence analysis in both 5637 and T24 BLCA cell lines (Figure 4E). To investigate the mechanism of PPARγ up-regulation after avasimibe treatment, qRT-PCR and luciferase reporter assay were used to detect the mRNA expression and the transcriptional activity of PPARγ, respectively. The results confirmed that the mRNA expression level and the transcriptional activity of PPARγ were significantly up-regulated after the avasimibe treatment (Supplementary Figure S4). The results confirmed that PPARγ mRNA expression level and transcription activity were significantly up-regulated after avasimibe treatment.

### GW9662, a PPARγ antagonist, reverted the cell cycle arrest induced by avasimibe in BLCA

**Figure 5:**
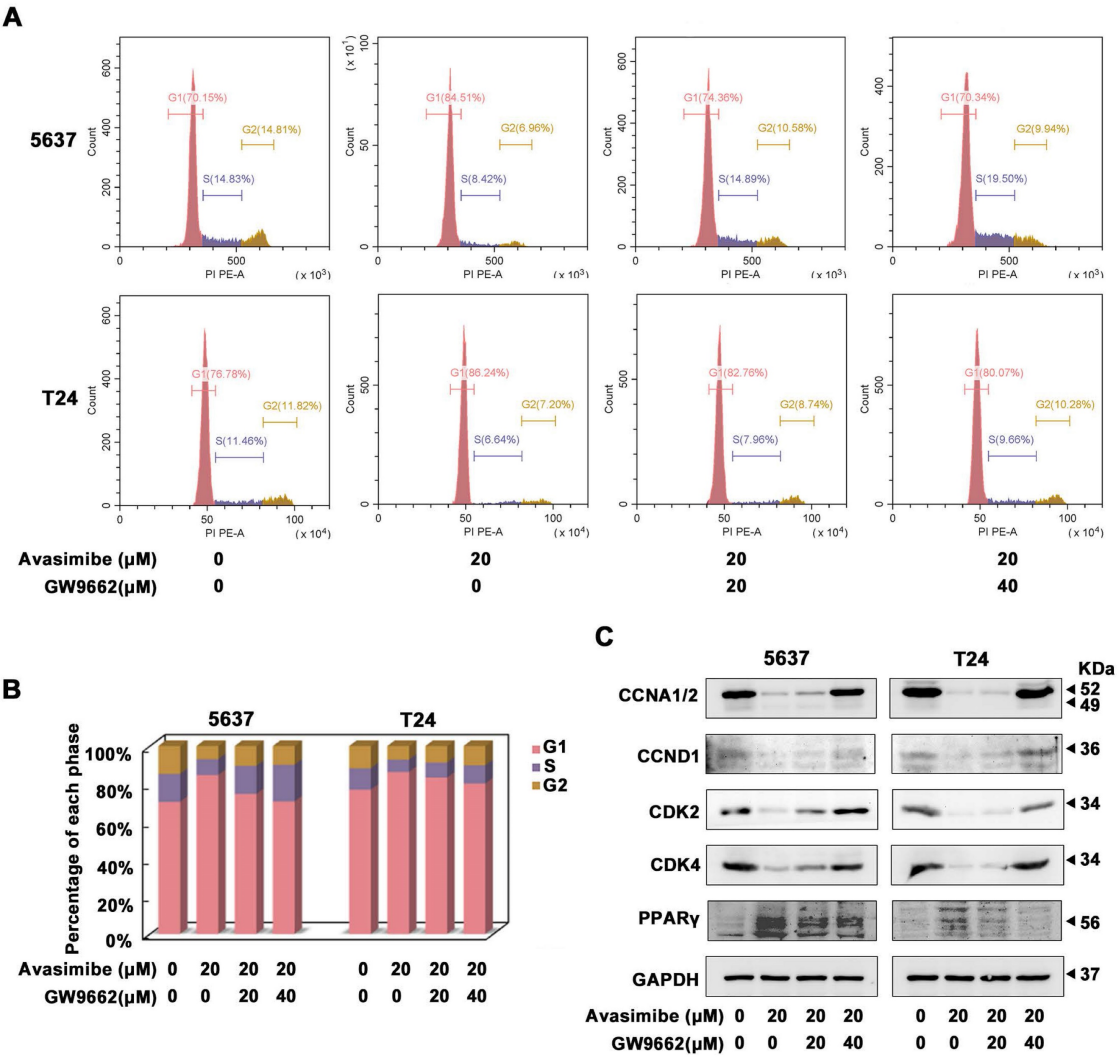
** GW9662, a PPARγ antagonist, reverted the cell cycle arrest induced by avasimibe in BLCA. (A-B)** The cell cycle distribution of BLCA cells after the combination treatment of avasimibe and GW9662 were analyzed by flow cytometry.** (C)** Content of cell cycle related proteins in BLCA cells were evaluated by Western blot analysis after the combination treatment of avasimibe and GW9662.

Two concentrations of GW9662, 20 μM and 40 μM, were used to investigate whether PPARγ was involved in the antitumor effects of avasimibe. As the Flow cytometry analysis showed, avasimibe-induced cell cycle arrest was significantly alleviated with increasing concentrations of GW9662 (Figure 5A-B). More notably, protein levels of CCNA1/2, CCND1, CDK2 and CDK4 were also restored significantly, after the combination of avasimibe and GW9662 (Figure 5C). However, GW9662 could not reverse the effect of avasimibe on the BLCA cells migration (Supplementary Figure S5).

### Avasimibe inhibited BLCA proliferation and metastasis *in vivo*

**Figure 6:**
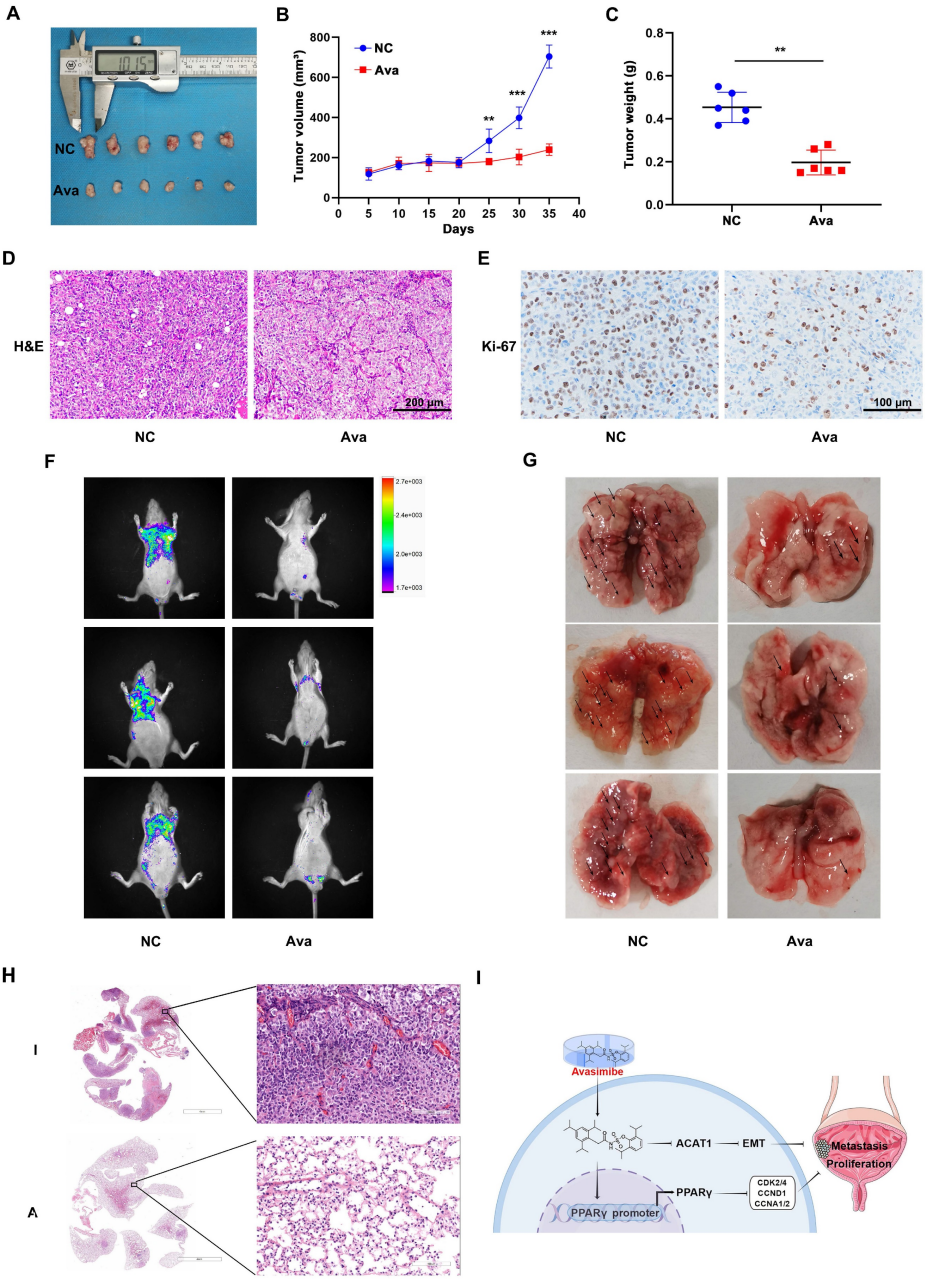
** Avasimibe inhibited BLCA growth and pulmonary metastasis *in vivo*. (A)** The xenograft model was established and the upper side is the control group while the lower side is the treatment group. **(B)** Tumor volume was calculated during the experiment.** (C)** After sacrificing, tumor weight of two groups was measured. **: *p* < 0.01; ***: *p* < 0.001. **(D-E)** H&E staining (D) and IHC staining of Ki-67 (E) were conducted on the tumor. **(F)** Fluorescence of pulmonary metastasis tumor was measured to evaluate the migration capacity. **(G)** Images of lungs removed from the mice. **(H)** H&E staining of mice lungs from different multiples of visual field.** (I)** Schematic illustration of the study.

A mouse model was established for further evaluating the effect of avasimibe on BLCA cell growth by subcutaneous transplantation of T24 cells (Figure 6A). The results showed that avasimibe could significantly inhibit tumor growth (Figure 6B). After 35 days of drug administration, all tumors were resected from mice. Results showed that the weight of tumor was significantly lower after the treatment of avasimibe (Figure 6C). Tumors were fixed and stained with H&E stating, and the result indicated that the cytological nuclear atypia and the nuclear-to-cytoplasmic ratio sharply decreased in the avasimibe group (Figure 6D). In addition, tumors were utilized for immunohistochemistry (IHC) staining of Ki-67 (Figure 6E). The results showed that Ki-67 positive cells in the avasimibe treatment group was significantly decreased, indicating that cell proliferation was strongly inhibited.

To investigate the effect of avasimibe on the metastasis of BLCA *in vivo*, pulmonary metastasis models were established. After tail vein injection of T24 cells, avasimibe was intraperitoneally injected for 4 weeks. We found that the fluorescence intensity of pulmonary metastases was significantly reduced after avasimibe treatment (Figure 6F). At the same time, the number of lung metastases lesions was obviously reduced after drug treatment (Figure 6G), which was further verified by the H&E stating of mice lungs (Figure 6H). These results suggested that avasimibe could inhibit BLCA progression *in vivo*.

## Discussion

Lipid homeostasis is necessary for mammalian cell growth. Cholesterol is an important component of cellular lipid species, which contains functional domains that regulate signal transduction and membrane surface lipids that affect membrane fluidity 5, 20. In recent years, many epidemiological studies have proved that cholesterol metabolism disorders are closely related to the occurrence, development, prognosis, and recurrence of tumors. In a prospective cohort study among 800,000 people analyzing metabolic factors in BLCA risk, BMI, cholesterol and triglycerides were shown to be relevant factors contributing to BLCA risk 21. In addition, it was reported that cholesterol intake was positively correlated with the incidence of BLCA, and the odds ratios was 1.54 (95% CI 1.14-2.08) for BLCA 22. The cytotoxicity of high intracellular cholesterol levels makes cholesterol esterification particularly important. Previous studies have demonstrated a strong link between cholesterol esterification and the development and progression of cancer 8, 23-25. ACATs play a key role in cholesterol metabolism, catalyzing the synthesis of cholesteryl esters from free cholesterol and fatty acyl-CoA 26. ACAT1 is one of two isoforms of ACAT and promotes the conversion of free cholesterol to cholesteryl esters 27. ACAT1 inhibitors have shown significant antitumor activity in a variety of *in vivo* and *in vitro* experiments 23, 28, 29. The regulatory function of ACAT1 has been studied in other cancer types, but its role in BLCA is still limited.

Avasimibe is an inhibitor of ACAT1 and has been used to treat atherosclerosis. In this study, we treated BLCA cells (5637 and T24) with different concentrations of avasimibe, and found that it had a significant BLCA inhibition effect in a dose-depend manner. The same results were also verified in the xenograft tumor models with nude mice. However, the corresponding concentration of avasimibe has no obvious inhibitory effect on the human uroepithelial cells SV-HUC-1 and human renal tubular epithelial cells HK2 (Supplementary Figure S1-S3). Various studies have shown that avasimibe can inhibit the metastasis of tumor cells 10, 30, 31. Consistently, our results showed that avasimibe significantly inhibited BLCA cells metastasis both in vivo and in vitro. EMT is closely related to tumor migration and is strictly regulated, which can make densely connected cells looser and more mobile, inducing cancer cells more prone to local infiltration and even distant metastasis. Meanwhile, it is widely believed that EMT involves alterations in gene expression and possesses several specific targets, such as E-cadherin, N-cadherin and Vimentin, etc. 32.

In this study, we found down-regulation of N-cadherin protein and up-regulation of E-cadherin protein in BLCA cells after avasimibe treatment. The cholesterol metabolism was closely related to the ROS metabolism, and it was reported that cholesterol could promote ROS generation by activating the NF-κB pathway 33. We observed an increase in ROS formation in avasimibe treated BLCA cells, suggesting that drugs can disrupt the homeostasis of ROS metabolism. SOD2 and catalase are antioxidant enzymes, which can effectively convert superoxide into hydrogen peroxide and further decompose it into water and oxygen, thereby protecting cells from oxidative damage induced by ROS 34, 35. We noticed that the protein expression levels of SOD2 and catalase were up-regulated in BLCA cells after avasimibe administration. Moreover, flow cytometry analysis showed that avasimibe induced cell cycle arrest at G1 phase. Taken together, we hypothesize that ACAT1 inhibition by avasimibe suppresses the proliferation and metastasis of BLCA cells, and that this effect may be related to disturbed cholesterol metabolism.

In our previous study, simvastatin treatment of BLCA cells resulted in a decrease in intracellular cholesterol content and inhibition of cell proliferation, invasion and migration, which may be caused by PPARγ signaling pathway 14. Treatment with ezetimibe showed that the viability of BLCA cells was inhibited and the cell cycle was arrested 36. The PPARs family PPARs consists of three isoforms, PPARα, PPARβ/δ and PPARγ, which are expressed at different expression levels in different tissues. Although they play different roles, some of their functions overlap. Studies have reported that they can interact with lipid metabolism pathways and can regulate the expression of genes involved in lipid metabolism 37, 38. We found that PPARγ was increased at both transcriptional and protein levels in BLCA cells after avasimibe treatment. We further used a combination of PPARγ antagonist GW9662 and avasimibe to treat BLCA cells, and found that GW9662 was able to restore the cell cycle arrest induced by avasimibe. However, cell migration ability was significantly inhibited.

Since the deep mechanism of BLCA inhibition by avasimibe remains unclear, the entry of avasimibe into real clinical antitumor applications remains to be further explored. The study of avasimibe in combination with other anti-neoplastic drugs for the treatment of breast cancer also provides more possibility for its clinical translational 39. In addition, although intraperitoneal administration of avasimibe, in DMSO with surfactant, has shown anti-tumor effects, this method is not suitable for clinical application 25, and needs to be further optimized.

In summary, our study reveals that ACAT1 was up-regulated in BLCA and was positively related to tumor grade. Avasimibe, an inhibitor of ACAT1, can attenuate BLCA tumorigenesis and induce G1-phase cell-cycle arrest by activating the PPARγ signaling pathway. Hence, inhibition of ACAT1 with avasimibe may provide a new idea for the therapeutic approach to BLCA.

## Supplementary Material

Supplementary figures and tables.

## Abbreviations

ACAT1: acyl-coenzyme A: cholesterol acyltransferase-1
BLCA: bladder cancer
DCFH-DA: 2',7'-Dichlorofluorescin diacetate
EMT: epithelial-mesenchymal transition
ER: endoplasmic reticulum
H&E: hematoxylin and eosin
IHC: immunohistochemistry
LDL: low-density lipoprotein
qRT-PCR: quantitative reverse transcription PCR
ROS: reactive oxygen species
