# Supplementary material for: Acyl-coenzyme A: cholesterol acyltransferase inhibitor avasimibe suppresses tumorigenesis and induces G1-phase cell-cycle arrest by activating PPARγ signaling pathway in bladder cancer
Source: J Cancer. 2024 Jan 1;15(2):370–82. doi: 10.7150/jca.83856 (PMC10758042; doi:10.7150/jca.83856)
Supplement: Supplementary file 1 — Supplementary figures and tables. [file jcav15p0370s1.pdf]

Supplementary materials

Supplementary Figures S1-S5

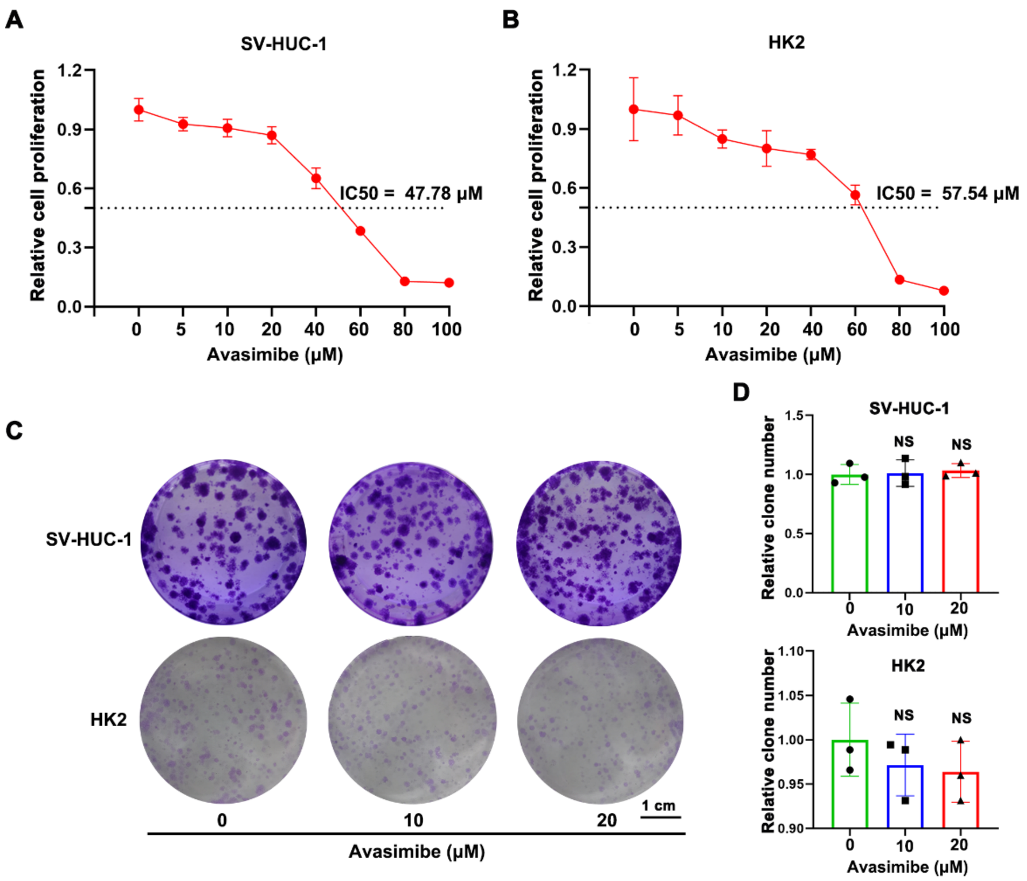

Supplementary Figure S1. MTT assay and clonogenic survival assay to test the viability and proliferation of cells treated with avasimibe.

(A-B) MTT assay was used to test the viability of SV-HUC-1 (A) and HK2 (B) treated with avasimibe. **(C-D)** Influence of avasimibe on cell survival was detected by clonogenic survival assay (C) and the statistical diagram (D), NS: not significant ($p > 0.05$).

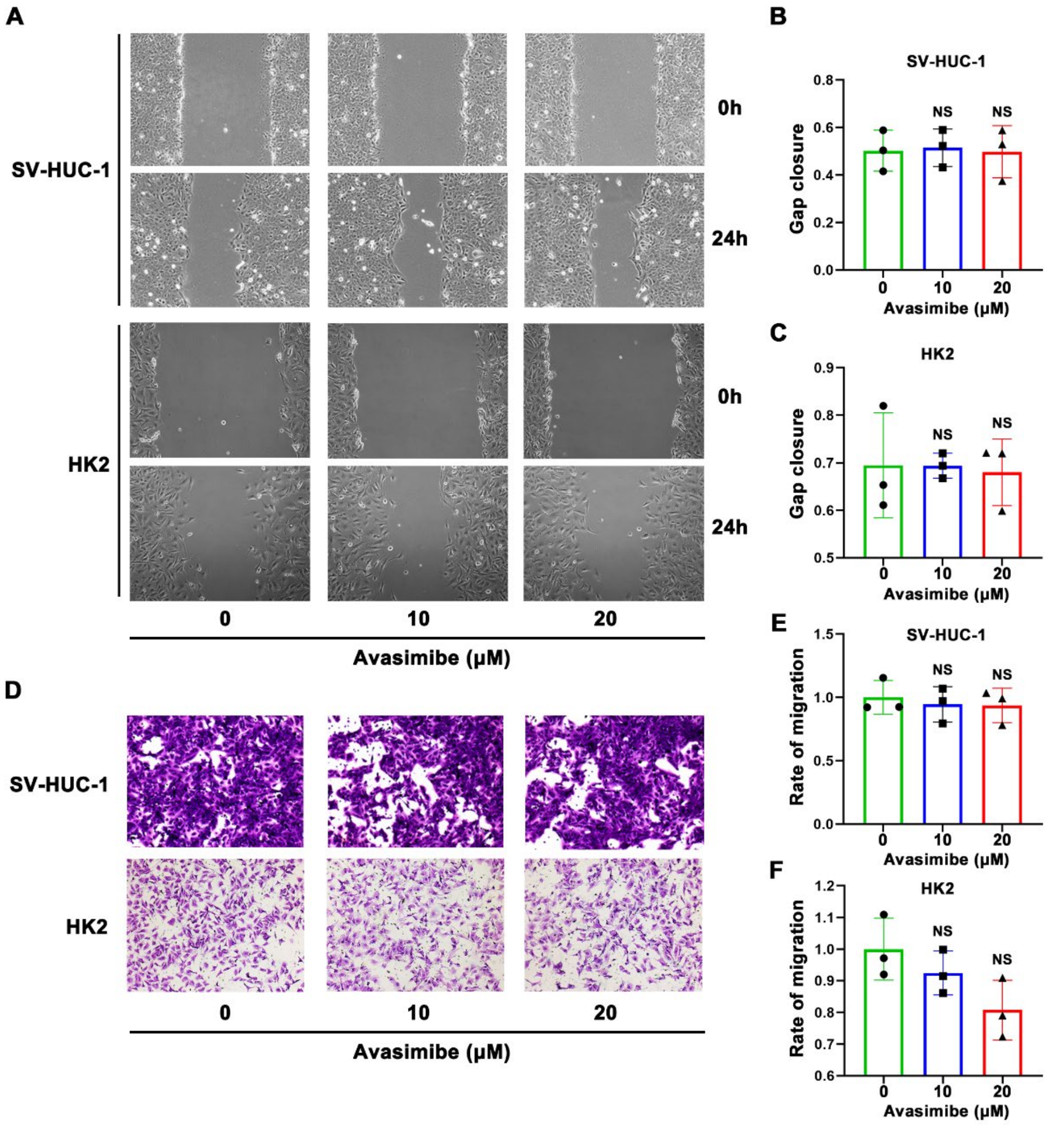

Supplementary Figure S2. Effect of avasimibe on the migration of SV-HUC-1 and HK2 cells.

(A-B) Wound healing assay was used to test the metastasis of BLCA after treated with avasimibe and the statistical diagram. **(C-D)** Effect of avasimibe on cell migration was detected by transwell chamber migration assay and the statistical diagram, NS: not significant ($p > 0.05$).

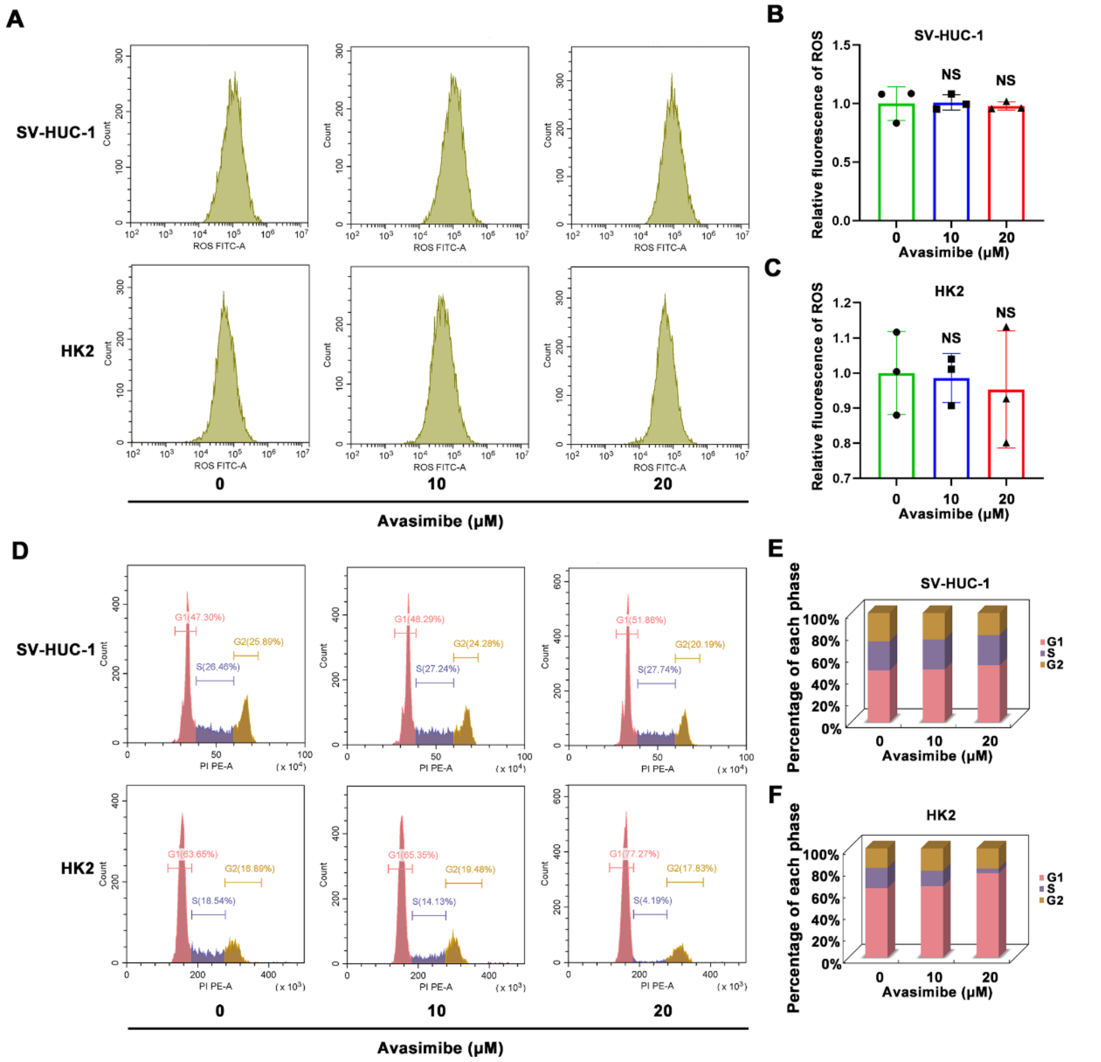

Supplementary Figure S3. Effect of avasimibe on cell cycle and ROS of SV-HUC-1 and HK2 cells.

(A-C) Production of ROS was detected by flow cytometry analysis and the statistical diagram, NS: not significant ($p > 0.05$). **(D-F)** Flow cytometry analysis of cell cycle after the treatment of avasimibe.

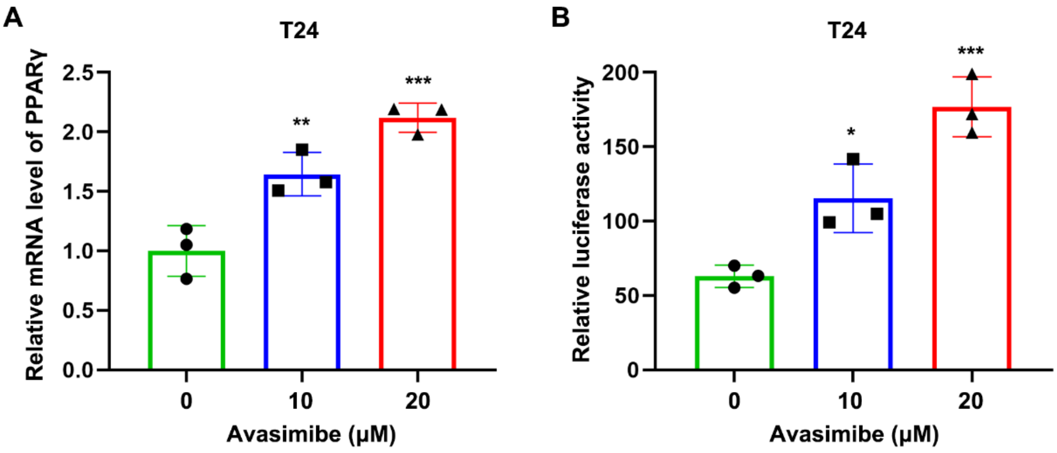

Supplementary Figure S4. Effects of avasimibe on the transcription activity of PPARγ.

(A) qRT-PCR detected the PPARγ mRNA after the treatment of avasimibe. **(B)** The luciferase reporter assay detected the PPARγ gene transcription activity after the treatment of avasimibe. *: $p < 0.05$; **: $p < 0.01$; ***: $p < 0.001$.

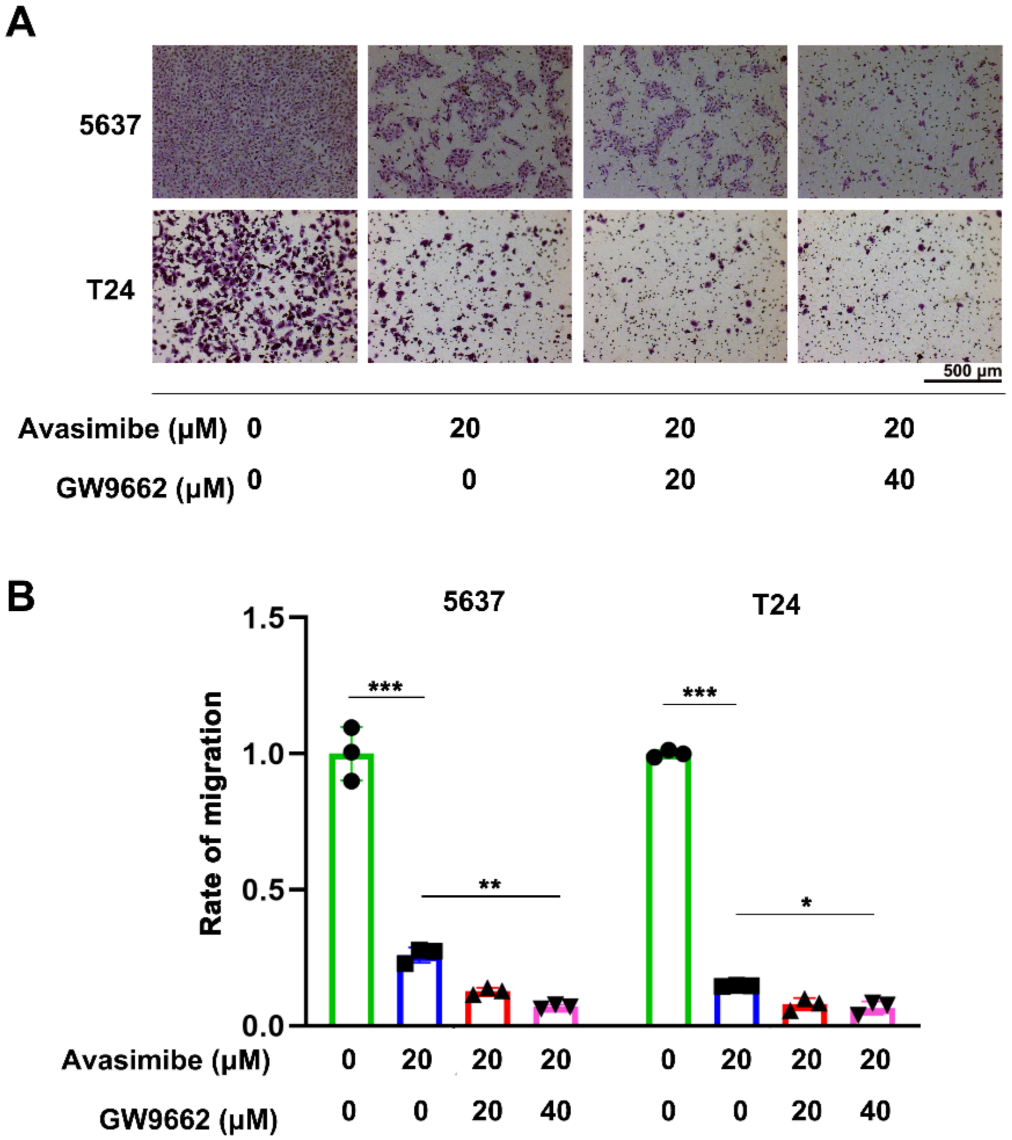

Supplementary Figure S5. GW9662 could not restore BLCA cells from avasimibe-induced migration inhibition.

(A) The influence of the combination treatment of avasimibe and GW9662 on cell migration was detected by transwell chamber migration assay and **(B)** is the statistical diagram, *: $p < 0.05$; **: $p < 0.01$; ***: $p < 0.001$.

Supplementary Tables S1-S3

Supplementary Table S1. Clinicopathological features of BLCA patients in the TCGA database.

Variables	Tumor (n = 411)	Normal (n = 19)
Age (year)	68.12 ± 10.59	69.89 ± 11.31
Gender [n (%)]		
Male	303 (26.28)	10 (47.37)
Female	108 (73.72)	9 (52.63)
Tumor stage [n (%)]		
I	2 (0.49)	0 (0.00)
II	131 (31.87)	4 (21.05)
III	141 (34.30)	7 (36.84)
IV	135 (32.85)	8 (42.11)
Unknown	2 (0.49)	0 (0.00)
T [n (%)]		
T0	1 (0.24)	0 (0.00)
T1	3 (0.73)	5 (26.32)
T2	120 (29.20)	11 (57.89)
T3	195 (47.45)	3 (15.79)
T4	59 (14.36)	0 (0.00)
Tx	1 (0.24)	0 (0.00)
Unknown	32(7.78)	0 (0.00)
N [n (%)]		
N0	239 (58.15)	11 (57.89)
N1	46 (11.19)	3 (15.79)
N2	76 (18.49)	5 (26.32)
N3	8 (1.95)	0 (0.00)
Nx	36 (8.76)	0 (0.00)
Unknown	6 (1.46)	0 (0.00)
M [n (%)]		
M0	196 (47.69)	10 (52.63)
M1	11 (2.68)	0 (0.00)
Mx	201 (48.90)	9 (43.37)
Unknown	3 (0.73)	0 (0.00)

Supplementary Table S2. Primary and secondary antibodies.

Antigen	Species source	Dilution (IF)	Dilution (WB)	Supplier
Ki-67	Mouse	1:200	-	Cell Signaling Technology, USA, Cat. #9449
GAPDH	Mouse	-	1:2000	Santa Cruz Biotechnology Inc., USA, Cat. #sc-365062
N-Cadherin	Rabbit	1:200	1:1000	Cell Signaling Technology, USA, Cat. #13116
E-Cadherin	Rabbit	1:200	-	Cell Signaling Technology, USA, Cat. #3195
Vimentin	Rabbit	-	1:1000	Cell Signaling Technology, USA, Cat. #5741
Slug	Rabbit	-	1:1000	Cell Signaling Technology, USA, Cat. #9585
SOD2	Rabbit	-	1:1000	ABLCAm，USA，Cat. #ab68155
Catalase	Rabbit	-	1:1000	ABLCAm，USA，Cat. #ab76024
Cyclin A1+A2	Rabbit	-	1:1000	ABLCAm，USA，Cat. #ab185619
Cyclin D1	Rabbit	-	1:1000	ABLCAm，USA，Cat. #ab16663
CDK2	Rabbit	-	1:1000	Cell Signaling Technology, USA, Cat. #2546
CDK4	Rabbit	-	1:1000	Cell Signaling Technology, USA, Cat. #12790
PPARγ	Rabbit	1:500	1:500	ABLCAm，USA，Cat. #ab45036
Anti-mouse IgG	Goat	-	1:5000	Sungene Biotech, China, Cat. # LK2003
Anti-rabbit IgG	Goat	-	1:5000	Sungene Biotech, China, cat. # LK2001
Anti-rabbit IgGF(ab')2 Fragment (Alexa Fluor® 488 Conjugate)	Goat	1:50	-	Cell Signaling Technology, USA, Cat. #4412
Anti-rabbit IgG (H+L), F (ab') 2 Fragment (Alexa Fluor 555 Conjugate)	Goat	1:50	-	Cell Signaling Technology, USA, Cat. #4413
Anti-mouse IgG (H+L), F(ab')2 Fragment (Alexa Fluor® 488 Conjugate)	Goat	1:50	-	Cell Signaling Technology, USA, Cat. #4407
Anti-mouse IgG (H+L), F(ab')2 Fragment (Alexa	Goat	1:50	-	Cell Signaling Technology, USA, Cat. #4408

Fluor® 555 Conjugate)				
Hoechst 3334 nucleic acid staining (DAPI)	-	1:750	-	Molecular Probes/Invitrogen, Carlsbad, CA, USA, Cat. #A11007

Supplementary Table S3. The primer sequences.

Gene	Forward primer (5'-3')	Reverse primer (5'-3')
GAPDH	GGAGCGAGATCCCTCCAAAAT	GGCTGTTGTCATACTTCCTCATGG
ACAT1	ATGCCAGTACACTGAATGATGG	GATGCAGCATATACAGGAGCAA
PPARγ	TACTGTCGGTTTTCAGAAATGCC	GTCAGCGGACTCTGGATTCAG
